# Classification of Different Fermentation Stages of Black Tea Using a Lightweight CNN Optimized by Knowledge Distillation

**DOI:** 10.3390/foods15101760

**Published:** 2026-05-15

**Authors:** Xuteng Liu, Mengqi Guo, Zhengtong He, Zhiwei Chen, Mei Wang, Chunwang Dong

**Affiliations:** 1 Tea Research Institute of Shandong Academy of Agricultural Sciences, Jinan 250100, China; 15964508016@163.com (X.L.); qq2524434302@163.com (M.G.); h_ztong@163.com (Z.H.); dongchunwang@163.com (C.D.); 2 Faculty of Engineering, Architecture and Information Technology, The University of Queensland, Brisbane, QLD 4072, Australia; 3 Shandong Academy of Agricultural Machinery Science, Jinan 250100, China

**Keywords:** fermentation classification, CNN model, lightweight, knowledge distillation, edge deployment

## Abstract

In red tea production, fermentation is critical for flavor. However, manual determination of its stages is inaccurate and inefficient, often spoiling flavor and lowering product value. To solve this, this study combines CNN and knowledge distillation to build a lightweight classification model, AT-ShuffleNet, for accurate, efficient stage identification in real processing. It collected images of Fuding and Tieguanyin tea at different fermentation stages. ResNet (teacher model) and ShuffleNet v2-0.5 (student model) were used for distillation. Focal and Poly Losses optimized both models to tap distillation potential. STD, MGD, SPKD, ATD, and KD methods were tested at various ratios to find the optimal strategy, forming AT-ShuffleNet. The lightweight model performed well: P (89.11%), R (90.16%), Kappa (89.29%), ACC (91.2%), F1 (89.53%). It addresses manual limitations, enabling accurate classification and reducing deployment issues in unstructured environments. For industrial validation, it was deployed on edge devices and integrated into a self-developed WeChat mini-program.

## 1. Introduction

Black tea, as one of the most popular beverages in the world [1], is favored by consumers for its unique flavor and rich nutritional value, accounting for approximately 75% of global tea product consumption. Black tea is produced from fresh tea leaves through four processing steps: withering, rolling, fermentation, and drying. Among these, fermentation is the key step that imparts the flavor to black tea [2,3,4]. After fermentation, polyphenols in the tea undergo oxidation and condensation with catechin as the substrate under the catalysis of polyphenol oxidase (PPO) and peroxidase (POD) [5], generating tea yellowine (TFs), tea redine (TRs), and a small amount of tea brownine (TBs). These oxidation products together constitute the unique flavor of black tea [6]. However, insufficient or excessive fermentation can significantly affect the quality of black tea. It is particularly important to accurately assess the fermentation stage during the processing. In traditional black tea processing, manual sensory judgment of the fermentation stage remains the mainstream method. However, this method lacks quantitative evaluation indicators and is susceptible to subjective factors such as processing environment, psychological state, and experience, leading to misjudgment of the fermentation stage and failing to meet the requirements of standardized production [7,8]. Therefore, establishing a precise and rapid method for assessing the fermentation stage has become a key task in the intelligent processing of black tea.

In recent years, researchers have explored the application of spectroscopy technology in determining the fermentation stage [9]. Spectroscopy technology can effectively capture the differences in spectral reflectance and be used to analyze the real-time changes in key chemical components such as water content, polyphenol content, and pigment concentration during the fermentation process. Mishra et al. used near-infrared hyperspectral imaging (HSI) combined with multiple-class SVM-ECOC models to rapidly and non-destructively classify six different types of commercial tea products [10], and the constructed classification model achieved a classification accuracy of 97.41% for tea products. Pinto et al. proposed a near-infrared (NIR) spectroscopy method combined with a partial least squares discriminant analysis (PLS-DA) model to rapidly identify the fermentation status and genetic type of 19 samples of Forastero cocoa beans [11]. These methods demonstrated excellent discrimination performance. However, spectroscopy equipment is expensive, data collection is complex, requires high environmental and computing resources, and is susceptible to environmental conditions, which limits its widespread application in practical situations.

The rapid development of deep learning algorithms has effectively solved these problems [12]. This technology can automatically extract and learn the complex features of fermented tea samples, enhancing the adaptability to fermentation states and environmental changes. This is of great significance for dealing with the dynamic and variable production conditions during actual processing. Kimutai et al. proposed the deep learning model TeaNet based on convolutional neural networks for automatic identification and evaluation of tea fermentation quality [13]. Compared with traditional machine learning algorithms such as RF, KNN, DT, SVM, LDA, and NB, TeaNet shows better performance in image classification tasks. Hendrawan et al. classified the quality grades of tempe (soybean fermented product) at different fermentation stages by using multiple convolutional neural network deep learning models [14]. Through training and testing of images of tempe samples at different fermentation stages, the classification accuracy of the model on tempe-fermented products reached 100% in the training set and 98.33% in the test set. Huang et al. combined hyperspectral imaging with multiple deep learning algorithms for non-destructive assessment of tea fermentation quality [15]. The results showed that deep learning methods significantly outperformed traditional machine learning algorithms in identifying the quality of black tea fermentation. Among them, the CNN-LSTM model optimized by PSO had the highest classification accuracy of 96.78% on the test set. However, most of these studies were designed for a single variety and lacked generalization ability. Moreover, as the performance of the model improved, the overly deep network structure and the huge parameter scale also limited its practical application in resource-constrained environments such as edge and mobile devices [16].

**Figure 1:**
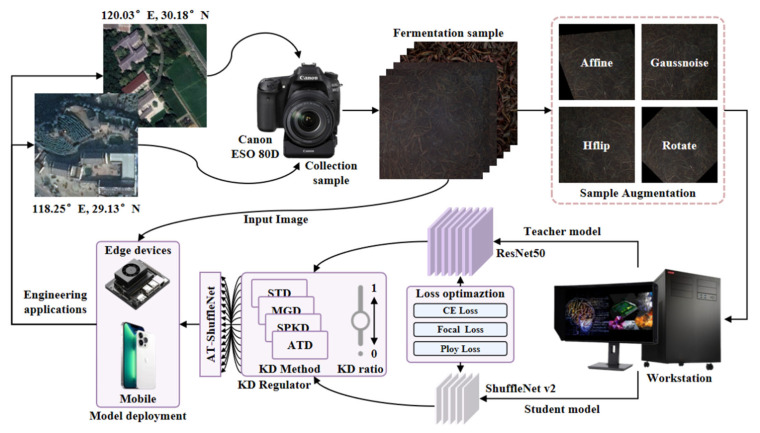
Research method flowchart.

In conclusion, although many scholars have proposed methods in the fermentation stage determination field that have achieved high accuracy, most existing approaches rely on high-complexity models that are unsuitable for deployment in resource constrained processing environments. Moreover, the potential of knowledge distillation in tea fermentation classification tasks remains underexplored. Most existing studies only adopt a single knowledge distillation strategy and rarely conduct systematic comparison, integration and optimization of multiple distillation schemes, which greatly limits the performance improvement potential of lightweight models in tea fermentation identification tasks. To address these limitations, this study collected fermentation images of different tea samples as the objects, combined with the CNN network and the knowledge distillation method, and designed a lightweight red tea fermentation classification model AT-ShuffleNet for the actual processing environment, aiming to achieve accurate and efficient fermentation stage discrimination in low computing power environments. The workflow is shown in Figure 1. The main contributions of this study are as follows:

(1) A dataset covering different fermentation stages of Fuding and Tieguanyin tea samples was constructed, encompassing six fermentation stage categories across two geographic locations and two seasonal collection periods. Various data augmentation methods were applied to enhance the model’s robustness and cross-variety generalization ability.

(2) Focal and Poly Losses were introduced to optimize the selected teacher model and student model to enhance the model’s discrimination ability for imbalanced or difficult-to-classify samples, and further explore the potential of model distillation within the knowledge distillation framework for tea fermentation classification.

(3) A systematic comparison of multiple knowledge distillation methods was conducted under varying KD ratios to identify the optimal distillation strategy for the tea fermentation classification task. Accordingly, the AT-ShuffleNet model was designed. While maintaining the model’s lightweight nature, the classification performance was significantly improved.

(4) Model deployment experiments were conducted on edge devices and our self-developed WeChat mini-program to verify the engineering application value and practical feasibility of the method, and the proposed model was compared with other lightweight models to validate its practical advantages.

## 2. Materials and Methods

### 2.1. Sample Collection

In this study, two tea tree varieties, Fudin and Tieguanyin, were selected. The fresh leaves of the tea were of one bud and one leaf. Fermentation experiments were conducted in September 2019 and July 2025 at the Tea Research Institute of the Chinese Academy of Agricultural Sciences (120.03° E, 30.18° N) and a tea culture park in Quzhou City, China (118.25° E, 29.13° N).

**Figure 2:**
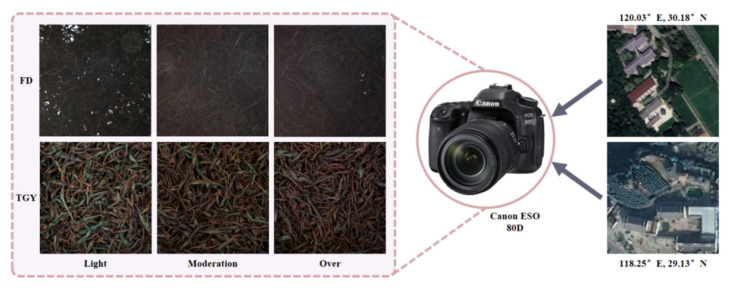
Sample collection process.

The fermentation experiments were conducted in a controlled LHS-150 artificial climate chamber (Peiyin experimental instrument Co., Ltd., Shanghai, China), where the temperature and relative humidity were strictly maintained at 30 °C and 90%, respectively, to provide an optimal and stable biochemical environment for enzymatic oxidation. During the fermentation process, to fully cover the fermentation stage of the black tea samples, the fermentation time of the experimental tea samples was extended to 5 h. The collection system consisted of a Canon EOS 80D single-lens reflex camera (Canon, Tokyo, Japan), a uniform light source, and a dark box. The system configuration, particularly the 40 cm distance between the camera and the samples, was optimized through preliminary experiments to achieve a balance between a sufficient field of view and high-resolution texture extraction while minimizing the potential thermal interference of the light source on the leaf surface. One sample was collected every hour, and a total of 390 images of black tea fermentation were collected. To make the model focus more on the fermentation state of the black tea, all images were cropped to square shapes to filter out the interference information at the image edges. Since this study was designed as an image classification task, no bounding-box or pixel-level annotation was required. Instead, each image was manually assigned to its corresponding fermentation-stage category and stored in the respective class folder as its label. Additionally, the two types of tea samples were numbered according to the fermentation stage and divided into six categories, FD-L, FD-M, FD-O, TGY-L, TGY-M, and TGY-O, representing the Light, Moderate, and Over Fermentation states of the Fuding and Tieguanyin tea samples, as shown in Figure 2. During the process, the visual characteristics of the tea leaves undergo a continuous transformation: the initial bright green or yellowish tones gradually shift toward a deeper reddish-brown as enzymatic oxidation progresses. In the later stages, particularly in the Over Fermentation category, there is a subtle but detectable decline in surface brightness and a transition toward more muted, darker hues. This evolutionary trend reflects the gradual change in organoleptic properties, where the Over Fermentation stage captures the onset of quality degradation often associated with excessive oxidation or heat accumulation in production. The dataset was divided into training, validation, and test sets in a 6:2:2 ratio.

### 2.2. Sample Augmentation

**Figure 3:**
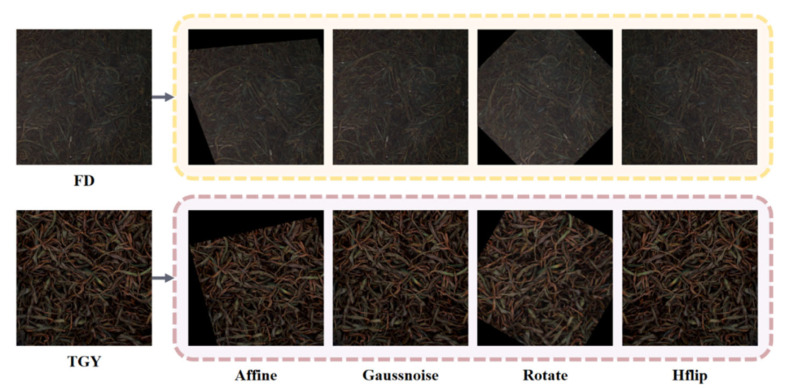
Display of samples after various augmentations.

Fuding and Tieguanyin have significant differences in appearance. If the model can simultaneously learn the color and texture evolution patterns of two representative tea samples, it can effectively enhance the model’s cross-variety generalization ability. However, due to the time-limited nature of the fermentation process, the original sample size is small, making it difficult to cover all uncontrollable factors’ disturbances. Therefore, in this study, we adopted data augmentation methods such as affine transformation, horizontal flipping, Gaussian noise, and random rotation to expand the divided dataset to further enhance the model’s robustness [17]. The sample collection process is shown in Figure 3.

### 2.3. Teacher and Student Model Architecture

In practical application scenarios, although deep convolutional neural networks can significantly enhance the recognition performance of the model, the continuous increase in the number of layers and parameters also brings huge computational and storage burdens, severely restricting the deployment and application of the model in resource-constrained environments such as edge devices and mobile terminals. To address this contradiction, this paper introduces knowledge distillation as an effective means of model compression and acceleration. By constructing a “teacher and student” model architecture, the rich knowledge contained in the large and high-performance teacher network is transferred to the more lightweight and efficient student network, thereby balancing model accuracy and deployment requirements. In terms of model selection, we combined experimental requirements and network characteristics, prioritizing the performance-optimized ResNet50 as the teacher model, and the compact and suitable for mobile deployment ShuffleNet v2-0.5x as the student model, providing a scientific and reasonable benchmark for subsequent distillation experiments.

#### 2.3.1. ResNet Model

**Figure 4:**
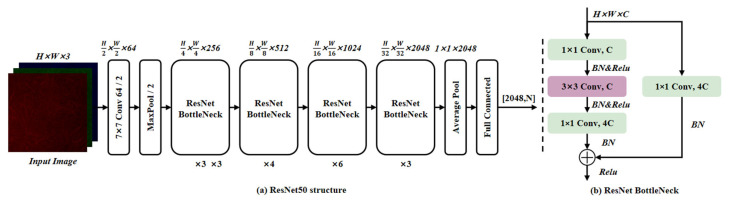
ResNet50 structure.

ResNet (Residual Network) is one of the most classic deep convolutional networks [18], as shown in Figure 4a of the architecture. Its core idea is to solve the training degradation problem of extremely deep networks using the residual learning approach. Before the proposal of the ResNet network, traditional convolutional neural networks were obtained by stacking a series of convolutional layers and downsampling layers. However, when stacked to a certain network depth, problems such as gradient vanishing or explosion and degradation would occur. To break through this bottleneck, ResNet proposed the residual structure, changing the traditional convolutional stacking to a residual block where the traditional convolution is combined with an identity jump. The input signal, in addition to passing through two layers of convolution, is directly added to the output through a shortcut path. This shortcut provides an additional channel for gradients during backpropagation, enabling extremely deep networks to be effectively optimized and avoiding degradation due to gradient vanishing or explosion. To balance depth and efficiency, the ResNet series gradually evolved into models tailored for different computational budgets. The earliest ResNet-18 and ResNet-34 were based on the residual idea and used two layers of 3 × 3 convolution to form a Basic Block, which was simple in structure and suitable for resource-constrained scenarios; when further deepened, the parameters and computational costs increased quadratically, so ResNet-50 and later versions introduced the Bottleneck design, as shown in Figure 4b, first reducing the channels through a 1 × 1 convolution, then extracting spatial features through a 3 × 3 convolution, and finally restoring the dimensions through a 1 × 1 convolution, thereby reducing the computational cost by approximately 4 times while almost not losing accuracy. Its excellent performance has made it a universal design paradigm for classification tasks.

#### 2.3.2. ShuffleNet v2 Model

**Figure 5:**
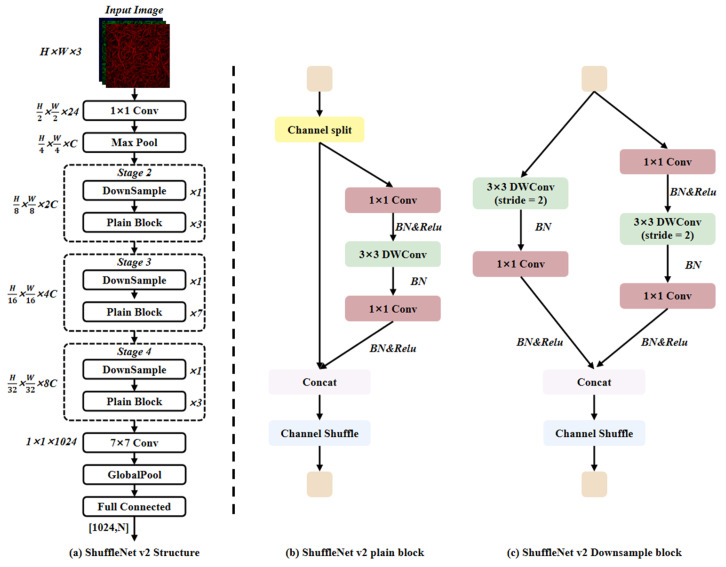
ShuffleNet v2 structure.

ShuffleNet v2 is an efficient convolutional network designed for mobile devices [19]. Its architecture is shown in Figure 5a and its core idea is to replace the traditional large kernel convolution with a combination of channel rearrangement and point-wise grouped convolution, thereby significantly reducing the computational cost while maintaining accuracy. Before the proposal of ShuffleNetV2, lightweight networks generally relied on depthwise separable convolution or bottleneck structures to compress parameters. However, when the number of channels and the width of the network further decreased, the memory access cost and fragmentation operations became new bottlenecks. To break through this limitation, ShuffleNetV2 constructed a Plain Block and Downsampling Block, as shown in Figure 5b. In each Block, the input features are first evenly divided into two branches along the channel dimension: one is directly passed as an identity mapping, and the other passes through 1 × 1 grouped convolution, 3 × 3 depth convolution, and 1 × 1 grouped convolution in sequence to extract features. Then, the two parts are concatenated along the channel dimension and shuffled by Channel Shuffle to achieve cross-group communication. This structure also provides additional gradient paths during backpropagation, which not only alleviates gradient disappearance but also reduces memory usage. To adapt to different computing platforms, the ShuffleNetV2 series has launched models with width multipliers of 0.5×, 1.0×, 1.5×, and 2.0×. Due to its highly lightweight characteristics, it is widely used in mobile visual classification tasks.

### 2.4. Loss Function

The commonly used CrossEntropy Loss in traditional classification models [20] can effectively guide the model’s learning process. However, it often performs poorly when dealing with class imbalance or difficult-to-classify samples. To further enhance the learning ability of the student model and better utilize the knowledge conveyed by the teacher model, this paper introduces improved loss functions such as Focal Loss and Poly Loss within the distillation framework. The aim is to achieve better knowledge transfer effects and model performance improvement by reasonably designing the loss function, thereby fully exploiting the potential of both the student and teacher models.

#### 2.4.1. Focal Loss

Focal Loss is a dynamic scaling-based CrossEntropy Loss [21]. In multi-classification tasks, when the class distribution is extremely imbalanced or easily distinguishable samples dominate, it enables the training to pay attention to the minority classes that are difficult to distinguish, thereby alleviating the easily distinguishable samples. Compared to the basic CrossEntropy Loss, Focal Loss incorporates a modulating factor (1−$P_{\mathrm{ic}}$)*γ* and class weights *α_c_*. The former reduces the loss contribution of well-classified samples, while the latter amplifies the gradient contribution of rare classes, thereby alleviating class imbalance.

(1) $$L_{\mathrm{FL}}=-\frac{1}{N}\sum_{i=1}^{N}\sum_{c=1}^{C}y_{\mathrm{ic}}\alpha_{c}{\left(1-p_{\mathrm{ic}}\right)}^{\lambda }\log p_{\mathrm{ic}}$$

#### 2.4.2. Poly Loss

Poly Loss reformulates the CrossEntropy Loss as a polynomial expansion, providing a more flexible form for loss optimization [22]. For each sample-category pair, the predicted probability $P_{\mathrm{ic}}$ is expanded as:

(2) $$-\log p_{\mathrm{ic}}=\sum_{k=1}^{\infty }\frac{{\left(1-p_{\mathrm{ic}}\right)}^{\lambda }}{\lambda }$$

Then, truncate to the first *m* items and assign trainable weights *ℇ_k_* to each item:

(3) $$L_{\mathrm{PL}}=-\frac{1}{N}\sum_{i=1}^{N}\sum_{c=1}^{C}y_{\mathrm{ic}}\left[\log p_{\mathrm{ic}}+\sum_{\lambda =1}^{m}\varepsilon_{\lambda }\frac{{\left(1-p_{\mathrm{ic}}\right)}^{\lambda }}{\lambda }\right]$$

### 2.5. Knowledge Distillation

Knowledge distillation [23] is a model compression technique. Its core idea is to transfer the knowledge learned by a large and complex teacher model to a simpler and smaller student model with fewer parameters. Specifically, during the training phase, the student model simultaneously fits the softened output distribution and the mid-level feature representations of the teacher model, using the combined loss of distillation and task losses for end-to-end optimization. Thus, while significantly reducing the number of parameters and computational complexity, it effectively enhances the representation ability and generalization performance of the student model.

#### 2.5.1. STD

**Figure 6:**
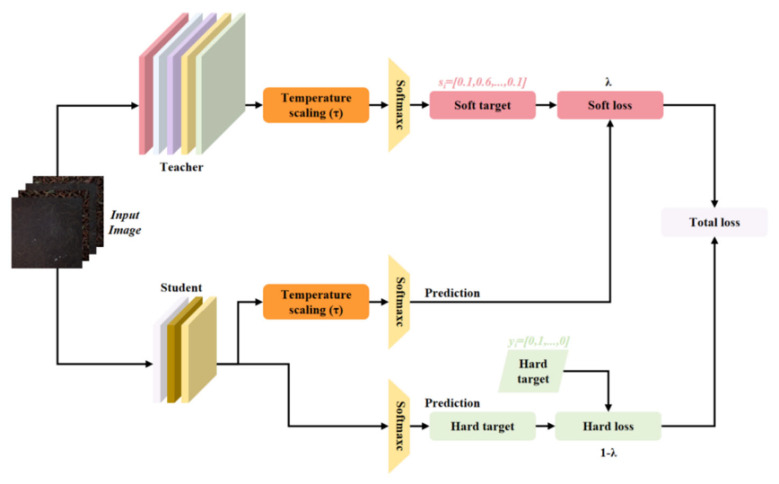
SoftTarget distillation strategy.

STD (SoftTarget Distillation) is the most classic and fundamental distillation strategy in knowledge distillation. Its core idea is to utilize the soft probability distribution of the teacher network after temperature scaling as an additional supervisory signal, enabling the student network to learn the latent knowledge of category similarity contained in the teacher model without relying on real hard labels, thereby achieving knowledge transfer and model compression, as shown in Figure 6. Specifically, STD adopts a temperature scaling and probabilistic imitation paradigm, where the teacher model generates the original logits $z_{i}^{T}$ for the input samples and softens them by introducing the temperature parameter *τ*.

(4) $$q_{i}^{T}=\frac{\exp\left(z_{i}^{T}/\tau \right)}{\sum_{j}z_{j}^{T}/\tau }$$

The high temperature τ amplifies the minor differences between logits, generating a smoother probability distribution and explaining the implicit relationships between classes. Meanwhile, the student network simultaneously generates the corresponding soft logits $z_{i}^{S}$, which are also softened by the temperature *τ*. The soft loss of both is minimized through KL divergence:

(5) $$L_{\mathrm{STD}}=\tau^{2}\cdot \mathrm{KL}\left(q^{T}∥q^{S}\right)=-\tau^{2}\sum_{i}q_{i}^{T}\log\frac{q_{i}^{S}}{q_{i}^{T}}$$

Here, *τ*^2^ counteracts the scaling effect of temperature on the magnitude of the gradient. Then, based on the actual Hard Target y, combined with *L_CE_* (CrossEntropy Loss) and distillation loss, the weights of hard labels and soft supervision are balanced through *λ*:

(6) $$L_{\mathrm{total}}=\alpha L_{\mathrm{CE}}\left(y,q_{T=1}^{S}\right)+\left(1-\alpha \right)L_{\mathrm{distill}}$$

#### 2.5.2. MGD

**Figure 7:**
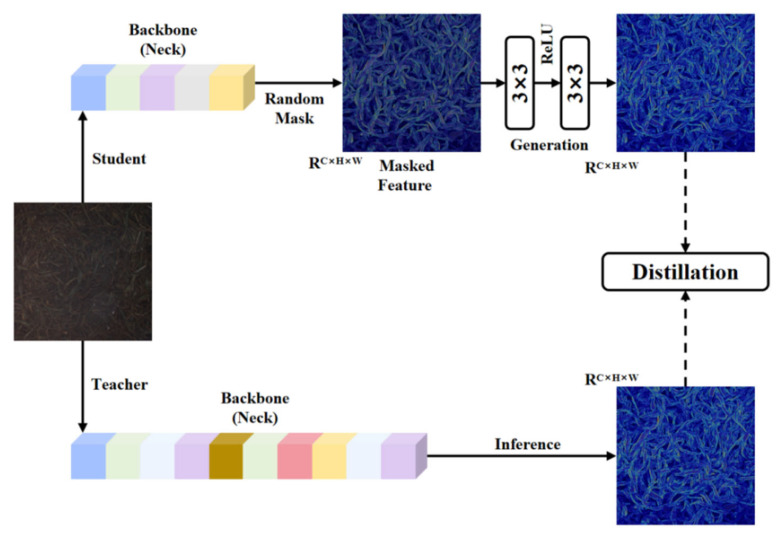
Masked Generative Distillation strategy.

MGD (Masked Generative Distillation) is a general knowledge distillation method based on features [24]. Its core idea is to mask some features of the student model and use a simple generation module to enable the student model to generate the complete features of the teacher model, thereby enhancing the representation ability of the student model without directly imitating the output of the teacher model. The specific implementation steps are shown in Figure 7. It aligns the channels of the student feature using 1 × 1 convolution, then randomly masks λ proportion of pixels to obtain the incomplete masked feature; subsequently, a lightweight generator G with two layers of 3 × 3 convolution uses these remaining pixels to restore the complete feature of the teacher. Finally, the mean square error of the restoration error is added as the distillation loss to the original task loss, forcing the student to still be able to infer the global structure in the case of local information loss.

#### 2.5.3. SPKD

**Figure 8:**
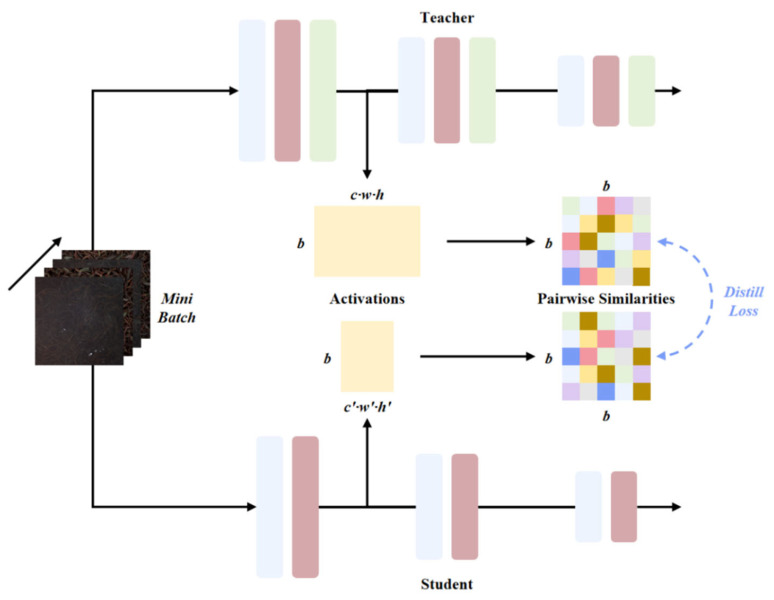
Similarity-Preserving Knowledge Distillation strategy.

SPKD (Similarity-Preserving Knowledge Distillation) is inspired by the observation that inputs with similar semantics often produce similar activation patterns in neural networks [25]. The core idea of SPKD is to enable similar activated inputs in the student network to generate the same activations as those in the teacher network, thereby guiding the learning of the student network. Specifically, SPKD calculates the pairwise similarity matrix of input samples and uses the difference in the similarity matrix between the student network and the teacher network for loss calculation. It aims to preserve the pairwise similarity relationships in the teacher network rather than directly imitating the representation space of the teacher network. As shown in Figure 8, SPKD forwards the input images through the teacher model T and the student model S, outputs the activation tensors, and reshapes the activation tensors into matrices $G_{\mathrm{ij}}^{T}$ and $G_{\mathrm{ij}}^{S}$. The loss function L_SPKD_ is calculated using the Frobenius norm to minimize the difference between the teacher and student similarity matrices. Finally, the total loss L_total_ is calculated by combining the CrossEntropy Loss, and the weights of the student network are updated through backpropagation to force it to maintain the sample similarity relationships in the teacher network.

#### 2.5.4. ATD

**Figure 9:**
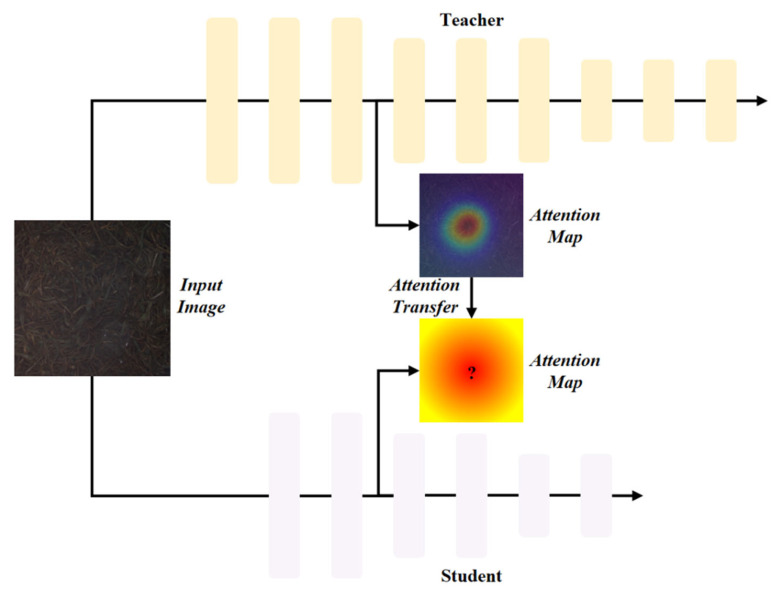
Attention Distillation strategy.

ATD (Attention Transfer Distillation) was the first to introduce the attention mechanism into the distillation method [26]. By forcing the student network to imitate the spatial attention distribution of the teacher network, it provides an efficient mechanism for knowledge transfer. The specific distillation process is shown in Figure 9. ATD defines attention as the spatial regions that the network focuses on during the decision-making process, which are mapped to the output activation tensor *A∈R^C×H×W^* of the convolutional layer. Statistical quantities are calculated along the channel dimension to generate the spatial attention map *F(A)∈R^H×W^*, and it is quantified as *Q^j^*. Then, the attention vectors $Q_{S}^{j}$ and $Q_{T}^{j}$ are *L2* normalized and aligned layer by layer for the loss. They are weighted and calculated together with the classification loss *L*_cls_ to obtain the total loss, and the student weights *W_S_* are updated through backpropagation to force them to simultaneously learn correct classification and the spatial attention distribution consistent with that of the teacher model:

(7) $$F^{j}\left(A\right)=\sum_{i=1}^{C}{\left|A_{i}^{j}\right|}^{p}$$

(8) $$Q^{j}=\mathrm{Vec}\left(F^{j}\left(A\right)\right)\in R^{HW}$$

(9) $$L_{\mathrm{AT}}=L_{\mathrm{cls}}\left(W_{s},x\right)+\frac{\beta }{2}\sum_{j\in L}{∥\frac{Q_{S}^{j}}{{∥Q_{S}^{j}∥}_{2}}-\frac{Q_{T}^{j}}{{∥Q_{T}^{j}∥}_{2}}∥}_{p}$$

## 3. Experiment Results and Analysis

### 3.1. Preparation of the Experiment

#### 3.1.1. Experimental Environment and Parameter Design

The training workstation used in this research is equipped with an Intel^®^ Core™ i7-14700K CPU and two NVIDIA GeForce RTX3090 24G GPUs in terms of hardware. Given that the classification work during the fermentation stage needs to be conducted in real time at the production line, multiple points need to be set up. If high-power GPU servers were equipped at each workstation, not only would the cabinet space be limited, but the cost would also be high, which does not conform to the actual working conditions. Therefore, this research selects the Jeston Orin NX 8GB as the model testing platform to simulate the actual working conditions. After the model training is completed, it is further migrated to the edge platform. Through the inference speed and accuracy tests under actual working conditions, the practical feasibility of the model is verified. Its hardware configuration consists of an 8-core ARM Cortex^®^-A78AE CPU and 1024 NVIDIA Core 32 Tensor Core 8G GPU. Both are programmed under the Ubuntu22.04 operating system using Visual Studio Code 1.119.0, and the virtual environment is jointly constructed by Python3.9, Torch2.3.1, and CUDA12.2 to ensure the compatibility of software and hardware.

During the training and testing phases, the hyperparameters of this study were set based on the classic combinations commonly used in the field of image classification [27,28]. The specific settings are shown in Table 1.

#### 3.1.2. Model Evaluation Indicators

To comprehensively evaluate the performance of the classification model, the paper refers to the general evaluation framework for classification tasks. For each category, based on the confidence level of the model’s output, different confidence threshold values are set to divide the prediction results. Then, Precision (P), Recall (R), Kappa coefficient, ACC (Accuracy), and F1 score are calculated successively. The calculation methods of each indicator are summarized as follows:

(10) $$P=\frac{\mathrm{TP}}{\left(\mathrm{TP}+\mathrm{FP}\right)}$$

(11) $$R=\frac{\mathrm{TP}}{\left(\mathrm{TP}+\mathrm{FN}\right)}$$

(12) $$p_{o}=\frac{\mathrm{TP}+\mathrm{TN}}{\mathrm{TP}+\mathrm{TN}+\mathrm{FP}+\mathrm{FN}}$$

(13) $$p_{c}=\frac{\left(\mathrm{TP}+\mathrm{FN}\right)\left(\mathrm{TP}+\mathrm{FP}\right)+\left(\mathrm{FP}+\mathrm{TN}\right)\left(\mathrm{FN}+\mathrm{TN}\right)}{{\left(\mathrm{TP}+\mathrm{TN}+\mathrm{FP}+\mathrm{FN}\right)}^{2}}$$

(14) $$\mathrm{Kappa}=\frac{p_{o}-p_{c}}{1-p_{c}}$$

(15) $$\mathrm{ACC}=\frac{\mathrm{TP}+\mathrm{TN}}{\mathrm{TP}+\mathrm{TN}+\mathrm{FP}+\mathrm{FN}}$$

(16) $$F1=2\times \frac{P\times R}{P+R}$$

Here, TP, TN, FP, and FN represent true positive, true negative, false positive, and false negative respectively.

Furthermore, in order to comprehensively evaluate the performance of the model, this study also introduced indicators such as Params (parameter), FLOPs (floating-point operations), and FPS (frames per second). These indicators can reflect the computational complexity and running efficiency of the model from different perspectives. The final performance evaluation of the model was completed on the test set.

### 3.2. Comparison Results of the Baseline

To verify the effectiveness of the teacher–student model architecture selected in this study, under the experimental conditions described in the “Preparation of the experiment” section, multiple classic and efficient CNN models were chosen for comparative experiments. Specifically, this study selected representative network architectures such as ResNet18, MobileNet v2, and DenseNet121 as the benchmark models to comprehensively evaluate the performance advantages of the selected models.

As shown in Table 2, ResNet50 achieved 87.33% in Kappa, 89.60% in ACC, and 87.93% in F1, significantly outperforming other models. This indicates that it possesses the highest cross-category consistency and comprehensive robustness under the condition of class imbalance. However, its parameter count and FLOPs reached 23.5 M and 4.09 G, only slightly lower than large models such as ResNet101, ConvNext-l, and VGG19. But as a teacher model, its performance is already excellent. Meanwhile, ShuffleNet v2-0.5x has only 0.34 M in parameter count and 0.04 G in FLOPs, with a reduction in computational cost by approximately two orders of magnitude. Its Kappa, ACC, and F1 indicators reached 84.32%, 87.20%, and 84.96% respectively. The model performance is still superior to large models such as DenseNet169 and the VGG series. This fully validates its potential as a lightweight student network. Therefore, by using ResNet50 as the teacher and ShuffleNet v2-0.5x as the student for knowledge distillation, the excellent performance of the teacher can be transferred to the student model with extremely low computational costs.

### 3.3. Selection of Loss Function

To further explore the potential of distillation, in this study, while maintaining the structure of the teacher and student models unchanged, the loss function was redefined. Focal Loss and Poly Loss were introduced as candidate strategies to obtain the optimal configuration of the teacher and student models with the highest distillation potential.

As shown in Table 3, ResNet50 still uses CrossEntropy as the loss function. This loss function significantly outperforms Focal and Poly in all five metrics: P, R, Kappa, ACC, and F1. Meanwhile, ShuffleNet v2-0.5x considers switching to Focal Loss. Compared to CrossEntropy Loss, this loss increases the P, R, Kappa, ACC, and F1 metrics by 0.75%, 3.01%, 1.64%, 1.2%, and 1.64% respectively, making it the optimal choice for this lightweight model.

### 3.4. Knowledge Distillation Strategy

To achieve the best distillation effect, this study selected the most classic and widely proven effective distillation methods such as STD, MGD, SPKD, and ATD. The KD ratio of these methods was adjusted, and comparative experiments were conducted under the same experimental conditions to select the optimal distillation strategy.

As shown in Table 4, compared with the student baseline model that has not undergone knowledge distillation, the four knowledge distillation methods show a significant distribution in performance. MGD performed the worst overall, and the performance of the distilled model was much lower than that of the NO KD model. This is likely because MGD was originally designed for dense prediction tasks such as object detection, and its masked feature generation mechanism is better suited to scenarios that require precise spatial feature alignment. For image-level classification tasks such as tea fermentation stage recognition, this feature alignment strategy does not match the task objective well, which limits its distillation effectiveness. SPKD performed well under all of the ratios while maintaining low computational cost and efficient recognition. It effectively improved the various indicators of the baseline model and was the most stable among all of the distillation methods. However, compared with the STD and ATD methods, the peak values of the indicators of the distilled model after using the SPKD method were lower. When the ratio was 0.4 and 0.8, the indicators of the STD and ATD distilled models reached their peaks, with P, R, Kappa, ACC, F1, etc., reaching 89.36, 88.17, 88.24, 90.4, 88.67, 89.11, 90.16%, 89.29%, 91.2%, 89.53%. Among them, ATD had the highest indicators for R, Kappa, ACC, F1, etc., when the ratio was set at 0.8, with only the P indicator slightly lower than that of STD-0.4, indicating that ATD achieved the best overall trade-off among the evaluated metrics and exhibited superior classification accuracy and recognition robustness. Therefore, ATD was selected as the final distillation strategy, and the resulting model was named AT-ShuffleNet.

### 3.5. Visualize Model Performance

#### 3.5.1. Confusion Matrix and Model Comparison

**Figure 10:**
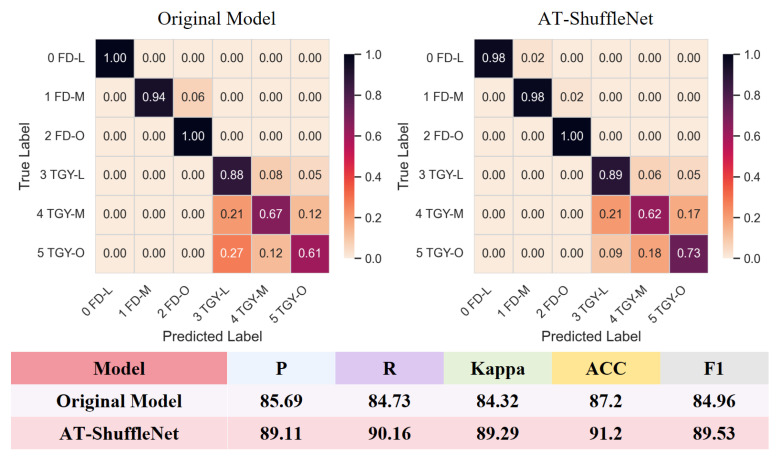
Comparison of confusion matrix and classification performance.

This study presents the confusion matrices and model comparisons of the original model and AT-ShuffleNet, as shown in Figure 10. The aim is to systematically compare the classification performance of both models for different varieties of tea samples during the fermentation stage. From the confusion matrices, it can be seen that the AT-ShuffleNet model has a significant advantage in identifying the fermentation stage samples of the less recognizable TGY tea samples. The number of misclassified samples of TGY-M and TGY-O has significantly decreased. Compared with the initial model, the ACC has increased by 12% and 18% respectively. The main sources of misclassification in the original model can be attributed to the high similarity in appearance and texture between adjacent fermentation stages of TGY, as well as insufficient extraction of subtle color and texture features. The attention mechanism in AT-ShuffleNet enables the model to focus on key fine-grained features related to fermentation degrees, thus effectively suppressing interference from similar appearances and reducing misjudgments. Moreover, compared with the initial model, the improved model has respectively increased the P, R, Kappa, ACC, and F1 indicators by 3.42%, 5.43%, 4.97%, 4%, and 4.57%. This indicates that the model has significantly improved in the identification of fine-grained features and complex samples during different fermentation stages.

#### 3.5.2. Comparative Analysis of t-SNE

**Figure 11:**
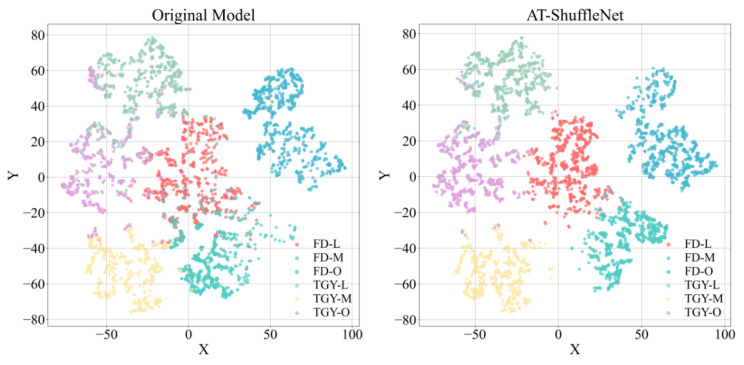
Strategy visualization of t-SNE classification performance.

t-SNE (t-Distributed Stochastic Neighbor Embedding) is a commonly used dimensionality reduction and visualization technique [29], mainly used to project high-dimensional data into a lower-dimensional space (usually 2D or 3D), making it easier to observe and analyze the structure and patterns of the data. To more clearly study the classification performance of the fermentation classification model before and after improvement, this study reduced the output dimensions of the original ShuffleNet v2-0.5 student model and AT-ShuffleNet to 2D, using them for visual analysis of the classification results. As shown in Figure 11, before the improvement, the clusters within different categories of tea samples at different fermentation stages were relatively scattered, and the confusion with other categories was relatively high, resulting in poor classification performance. However, the improved model presented different distributions, not only with more compact internal distribution of clusters, but also with better optimization of the confusion between different categories. This indicates that AT-ShuffleNet has better classification ability for different tea sample fermentation stages and can more accurately determine the fermentation state.

### 3.6. Model Deployment and Programming

To verify the engineering practicality and edge-side adaptability of the proposed method [30], this study not only deployed the trained AT-ShuffleNet model but also introduced multiple representative lightweight models for synchronous cross-platform deployment and comparison tests. The trained models in the PyTorch framework were uniformly converted to ONNX format and tested on the edge device platform described in the experimental setup, with detailed results presented in Table 5.

As can be seen from the table, the classification performance metrics including precision, recall, Kappa, accuracy and F1-score of all the models remain completely consistent between workstations and edge devices, with no performance degradation observed after deployment. This is attributed to the fixed network structure and learned model weights, which ensure stable recognition results across different hardware platforms. Meanwhile, the FPS values differ significantly between the two platforms, which is a normal objective phenomenon caused by the substantial gap in computing power and inference performance between high-performance workstations and resource-constrained edge devices.

Among all the compared models, the AT-ShuffleNet still maintains outstanding classification performance on edge devices, with P, R, Kappa, ACC and F1 reaching 89.11%, 90.16%, 89.29%, 91.2% and 89.53% respectively, showing no loss in accuracy compared with the workstation test results. Meanwhile, its FPS on edge devices remains 203.18, which fully meets the real-time processing demands of actual tea fermentation detection scenarios. The above results fully demonstrate the strong engineering feasibility and practical application value of the proposed AT-ShuffleNet model in real industrial environments.

**Figure 12:**
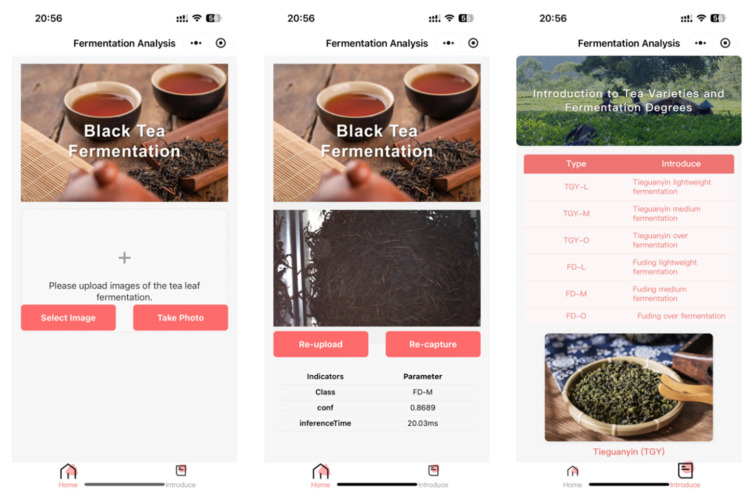
Black tea fermentation identification applet.

Furthermore, considering that the deployment of deep learning models on edge devices still requires additional hardware configuration and corresponding cost investment, which limits its popularization and application in actual tea production scenarios, this study further developed a WeChat Mini Program for intelligent identification of tea fermentation stages to achieve lightweight, portable and user-friendly detection. The system is constructed based on the ONNX Runtime deep learning inference framework and Flask back-end application architecture, and relies on Tencent Cloud server resources to complete model deployment, image processing and result return. As shown in Figure 12, the overall structure of the mini-program is clear and stable, which mainly includes two core functional modules: the Home page module and Fermentation knowledge introduction module.

In the Home module, users can upload tea fermentation sample images through two flexible ways: selecting local images from the mobile phone album or calling the camera to take real-time photos on the production site. After the image is uploaded successfully, the system will automatically perform preprocessing such as size normalization and channel adjustment, then call the lightweight AT-ShuffleNet inference model deployed on the cloud server to complete real-time prediction and reasoning, and quickly return the specific fermentation stage, confidence score and corresponding judgment basis to the user interface. The running test of the mini-program was carried out on an iPhone 13 smartphone, and the average single-image detection speed was stable at approximately 20–40 ms, which meets the requirements of rapid identification in actual scenes. Through this friendly and convenient human–computer interaction mode, users can quickly obtain accurate and intuitive feedback on the fermentation degree of different tea samples without professional equipment, truly realizing portable, low-cost and widely applicable intelligent tea fermentation detection.

The Introduce module is designed to provide users with comprehensive and systematic tea fermentation science popularization services. It not only elaborates the appearance characteristics, color changes, texture differences and aroma performance corresponding to each fermentation grade in detail, but also systematically explains the intrinsic correlation between different tea categories and their suitable fermentation processes and stages, helping users deeply understand the scientific mechanism, key influencing factors and quality control logic of tea fermentation, so as to improve the practical guiding significance of the system in tea production and processing.

## 4. Conclusions

This study combines the CNN model and the knowledge distillation method to propose a lightweight tea fermentation classification model named AT-ShuffleNet for practical processing environments. ResNet and ShuffleNet v2-0.5 are selected as the teacher and student models for the distillation experiments respectively; Focal and Poly Losses are adopted for optimization to explore the optimal distillation potential; STD, MGD, SPKD, and ATD distillation methods are introduced to conduct distillation experiments for different KD ratios, aiming to explore the optimal distillation strategy while keeping the student model lightweight and achieving accurate discrimination of different varieties of tea during the fermentation stage.

The experimental results show that the ShuffleNet v2-0.5 model, optimized by the loss function and distilled with the optimal strategy, maintains a lightweight volume of only 0.34 M parameters and 0.04 G FLOPs. Meanwhile, all of the performance indicators have been significantly improved compared to the original ShuffleNet v2-0.5 model. Specifically, P, R, Kappa, ACC, and F1 have increased by 3.42%, 5.43%, 4.97%, 4%, and 4.57% respectively, reaching 89.11%, 90.16%, 89.29%, 91.2%, and 89.53%. This effectively proves the reliability and effectiveness of the proposed tea fermentation classification model. Additionally, this study deployed the tea fermentation classification model on edge devices and self-developed WeChat mini-programs to demonstrate the feasibility of this method in edge devices and mobile applications, and to verify its practical significance in an engineering environment.

It is worth noting that this study only used two types of tea samples, namely Fuding tea and Tieguanyin tea, as the research objects. Although this effectively improved the generalization performance of the model, it still has limitations. Since the enzymatic oxidation during fermentation follows a consistent logic of color and luster evolution, the proposed framework possesses the potential to be migrated to other tea varieties through fine-tuning. Future work will collect tea samples from different seasons and varieties to establish a more complete database of fermentation samples, in order to enhance the model’s generalization ability for different tea samples and working conditions. On the other hand, this study used RGB images as experimental data, mainly learning the visual characteristics of fermented tea samples. Specifically, the model captures the continuous transformation of color from bright to deep and the subtle decay of surface luster, which are key visual indicators of fermentation depth and potential quality degradation. It did not deeply capture the changes in the chemical components of the tea samples and lacked an understanding of the chemical mechanism of the tea samples, which limited the model’s comprehensive understanding of the fermentation process. Therefore, relying solely on visual information may be insufficient to fully characterize internal quality attributes like aroma and taste. Future work will also integrate spectral data or other sensory indicators to build a multimodal database, in order to enhance the model’s understanding of the complex fermentation process and improve the reliability of quality anomaly detection.

## Figures and Tables

**Table 1:** Hyperparameters design.

Name	Description	Values
lr	Learning rate	0.001
lrs	Learning rate scheduler	CosineAnnealingLR
Momentum	SGD momentum/Adam beta1	0.9
Optimizer	Optimizer to use	Adam
Min lr	Min learning rate	0.000001
workers	Data-loading threads	4
Image size	Input images size	640 × 640
Batch size	Number of images per batch	16
Training epochs	Baseline training epochs	300
Distill epochs	Model distillation training epochs	300
Loss	Loss function	CrossEntropy

**Table 2:** Comparison results of the baseline.

Model	P (%)	R (%)	Kappa (%)	ACC (%)	F1 (%)	Params (M)	FLOPs (G)	FPS
ResNet18	85.86	85.11	84.36	87.2	85.31	11.18	1.81	828.06
ResNet34	86.37	85.04	84.78	87.6	85.48	21.28	3.66	592.85
**ResNet50**	**88.25**	**88.29**	**87.33**	**89.6**	**87.93**	23.50	4.09	462.14
ResNet101	86.48	86.62	85.35	88.0	86.31	42.46	7.81	245.70
**ShuffleNet v2-0.5x**	85.69	84.73	84.32	87.2	84.96	**0.34**	**0.04**	**963.47**
ShuffleNet v2-1.0x	85.09	83.56	84.78	87.6	83.98	1.25	0.14	932.83
MobileNet v2	88.02	86.68	86.34	88.8	86.80	2.21	0.30	845.55
MobileNet v3	85.52	84.28	84.31	87.2	84.77	4.20	0.22	805.61
DenseNet121	85.40	84.78	83.45	86.4	84.24	6.92	2.89	202.95
DenseNet161	85.54	85.36	84.36	86.8	85.32	12.49	3.43	144.92
DenseNet169	84.89	84.02	83.83	87.2	84.23	26.49	7.84	131.61
DenseNet201	84.99	82.87	83.32	86.4	83.51	18.10	4.39	126.49
VGG11	83.05	83.69	82.48	85.6	83.24	128.79	7.61	386.95
VGG13	83.76	83.51	82.95	86.0	83.39	128.98	11.30	287.86
VGG16	84.15	83.91	83.01	86.4	83.77	134.29	15.47	239.47
VGG19	84.28	84.22	83.28	86.0	84.34	139.59	19.63	197.65
EfficientNet v2-s	85.43	84.91	83.93	86.8	84.77	20.11	2.85	381.74
EfficientNet v2-m	87.15	85.51	85.27	88.0	85.95	52.72	5.37	263.60
EfficientNet v2-L	85.48	84.81	83.71	86.6	84.60	116.99	12.24	106.94
ConvNext-t	76.97	75.04	74.40	79.2	73.86	27.80	4.45	257.17
ConvNext-s	76.34	76.55	74.67	79.2	75.48	49.42	8.68	133.02
ConvNext-l	76.00	75.25	71.76	82.0	72.48	196.16	34.36	64.85
GhostNet	87.72	86.79	85.85	88.4	86.80	3.90	0.14	588.80

**Table 3:** Loss function optimization results.

Model	Loss	P (%)	R (%)	Kappa (%)	ACC (%)	F1 (%)	FPS
**ResNet50**	CrossEntropy	88.25	88.29	87.33	89.6	87.93	462.14
	Focal	87.24	88.24	86.86	89.2	87.58	388.67
	Poly	88.35	85.52	85.77	88.4	86.40	393.84
**ShuffleNet v2-0.5x**	CrossEntropy	85.69	84.73	84.32	87.2	84.96	963.47
	Focal	86.44	87.74	85.96	88.4	86.60	929.65
	Poly	85.71	86.51	84.94	87.6	85.70	961.47

**Table 4:** Distillation comparative experiment.

KD Strategy	Loss Ratio	P (%)	R (%)	Kappa (%)	ACC (%)	F1 (%)
NO KD	/	86.44	87.74	85.96	88.4	86.60
STD	0.1	86.14	86.06	86.32	88.8	86.08
	0.2	89.39	86.12	87.20	89.6	87.21
	0.3	87.70	88.92	87.39	89.6	87.89
	0.4	**89.36**	88.17	88.24	90.4	88.67
	0.5	86.88	86.88	86.81	89.2	86.84
	0.6	85.45	86.01	84.46	87.2	85.24
	0.7	86.88	87.93	86.87	89.2	87.23
	0.8	85.86	86.08	85.85	88.4	85.91
	0.9	85.52	86.99	84.97	87.6	85.80
MGD	0.1	83.40	84.38	82.57	85.6	83.45
	0.2	80.77	81.01	80.13	83.6	80.54
	0.3	79.46	79.65	77.71	81.6	78.93
	0.4	74.24	73.87	72.74	77.6	73.85
	0.5	73.77	74.49	72.66	77.6	72.98
	0.6	77.91	74.46	70.85	76.0	70.23
	0.7	70.24	70.53	69.77	75.6	66.64
	0.8	75.80	70.43	69.79	75.6	69.56
	0.9	70.60	70.18	69.53	69.6	66.36
SPKD	0.1	88.29	87.40	87.76	90.0	87.73
	0.2	89.04	90.03	88.81	90.8	89.31
	0.3	86.18	86.05	86.05	89.2	86.10
	0.4	86.84	87.82	86.39	88.8	87.16
	0.5	88.24	87.82	87.77	90.0	88.01
	0.6	86.27	87.14	86.39	88.8	86.54
	0.7	87.73	88.36	87.82	90.0	88.00
	0.8	87.51	87.98	87.81	90.0	87.68
	0.9	88.56	89.68	88.32	90.4	88.88
ATD	0.1	87.72	88.19	86.68	89.2	87.42
	0.2	87.74	87.87	87.83	90.0	87.61
	0.3	85.75	87.02	85.43	88.0	86.17
	0.4	86.38	87.30	86.40	88.8	86.56
	0.5	85.57	86.57	84.96	87.6	85.72
	0.6	88.44	89.58	87.84	90.0	88.85
	0.7	86.35	85.20	86.27	88.8	85.55
	0.8	89.11	**90.16**	**89.29**	**91.2**	**89.53**
	0.9	87.95	88	87.33	89.6	87.8

**Table 5:** Edge deployment performance.

Performance	Model	P (%)	R (%)	Kappa (%)	ACC (%)	F1 (%)	FPS
**Workstation**	ResNet18	85.86	85.11	84.36	87.2	85.31	828.06
	MobileNet v2	88.02	86.68	86.34	88.8	86.80	845.55
	MobileNet v3	85.52	84.28	84.31	87.2	84.77	805.61
	ResNet50	88.25	88.29	87.33	89.6	87.93	462.14
	GhostNet	87.72	86.79	85.85	88.4	86.80	588.80
	AT-ShuffleNet	89.11	90.16	89.29	91.2	89.53	929.65
**Edge**	ResNet18	85.86	85.11	84.36	87.2	85.31	178.52
	MobileNet v2	88.02	86.68	86.34	88.8	86.80	182.37
	MobileNet v3	85.52	84.28	84.31	87.2	84.77	174.91
	ResNet50	88.25	88.29	87.33	89.6	87.93	102.76
	GhostNet	87.72	86.79	85.85	88.4	86.80	125.43
	AT-ShuffleNet	89.11	90.16	89.29	91.2	89.53	203.18

## Data Availability

The data presented in this study are available on request from the corresponding author due to the authors do not have permission to share the data.
